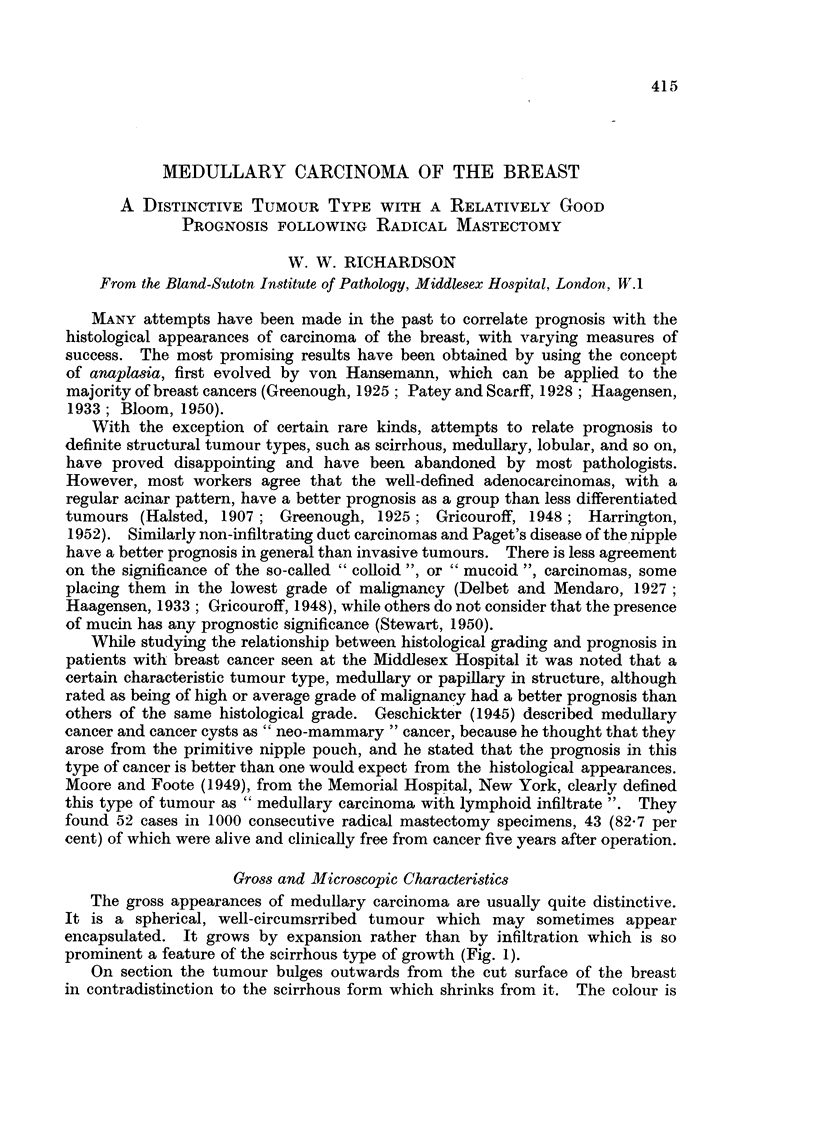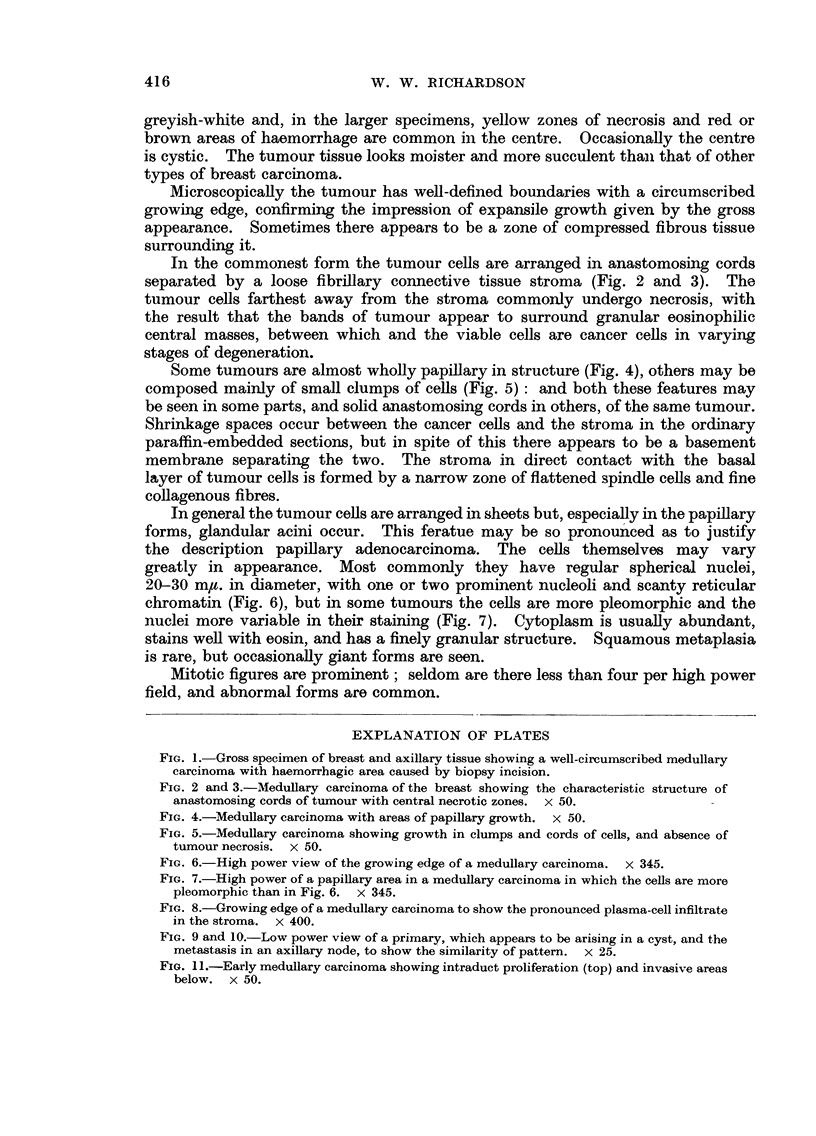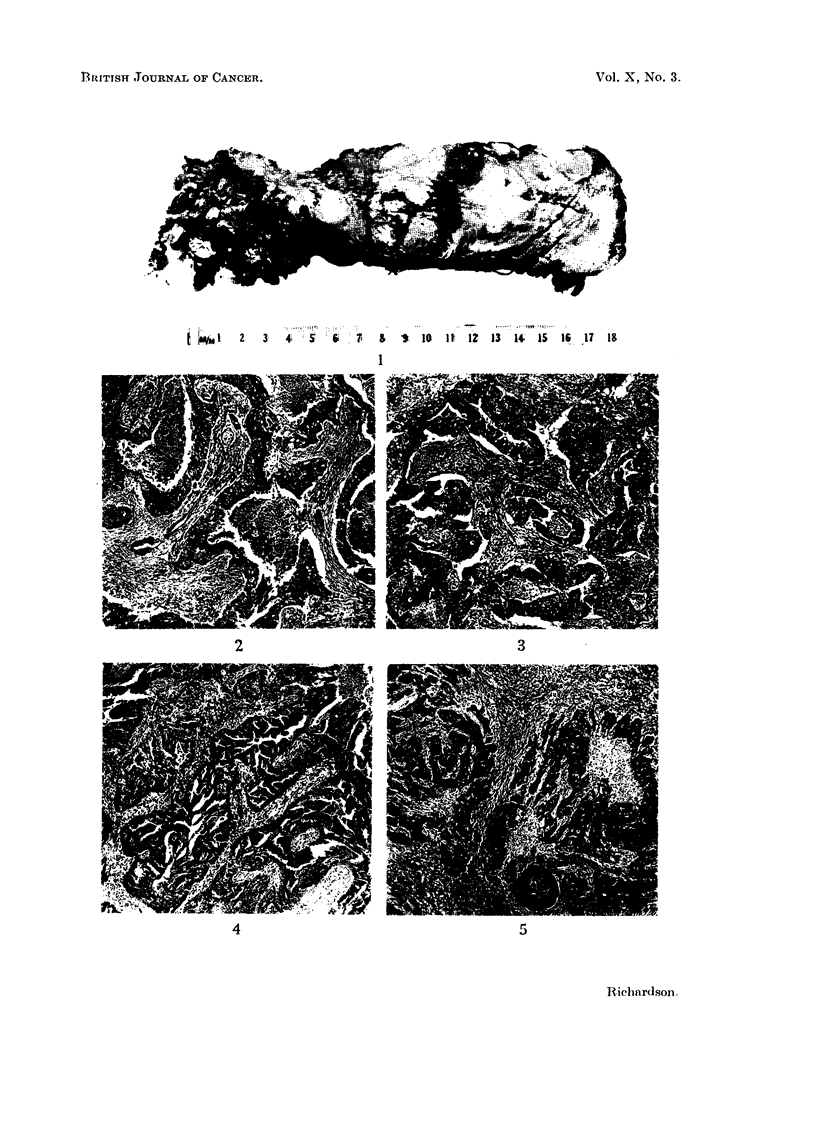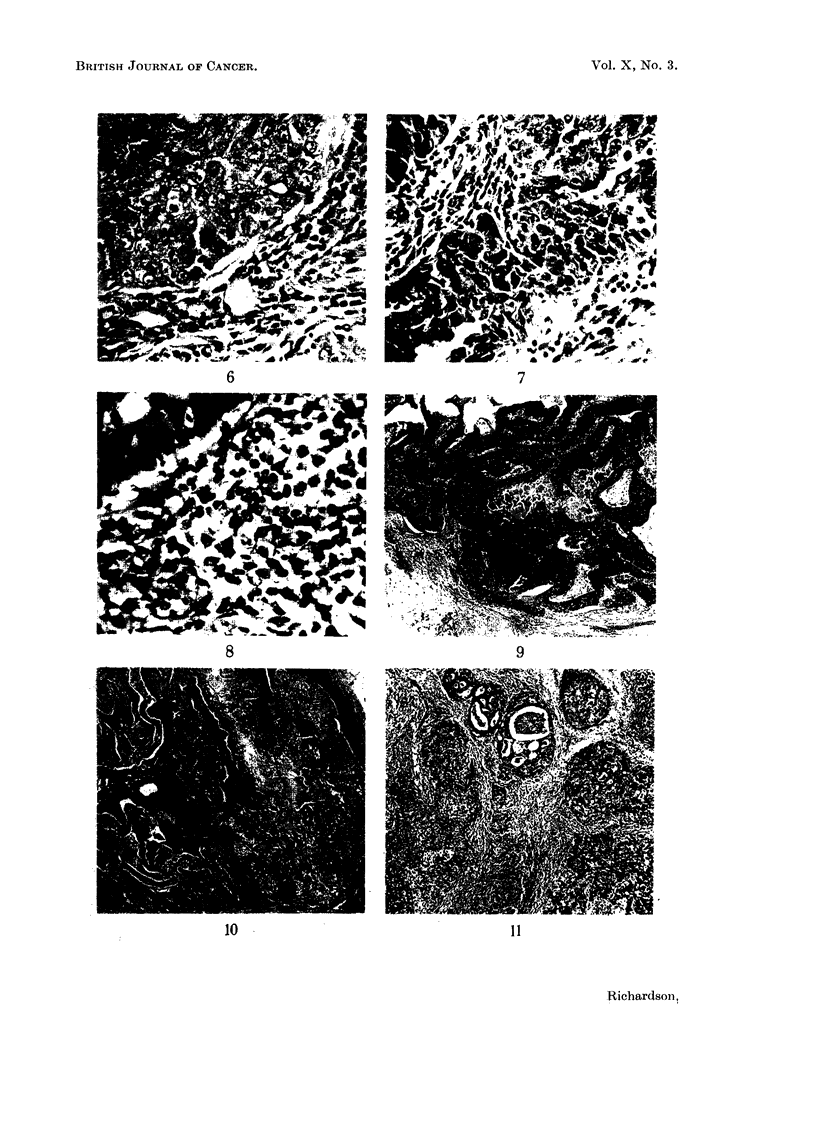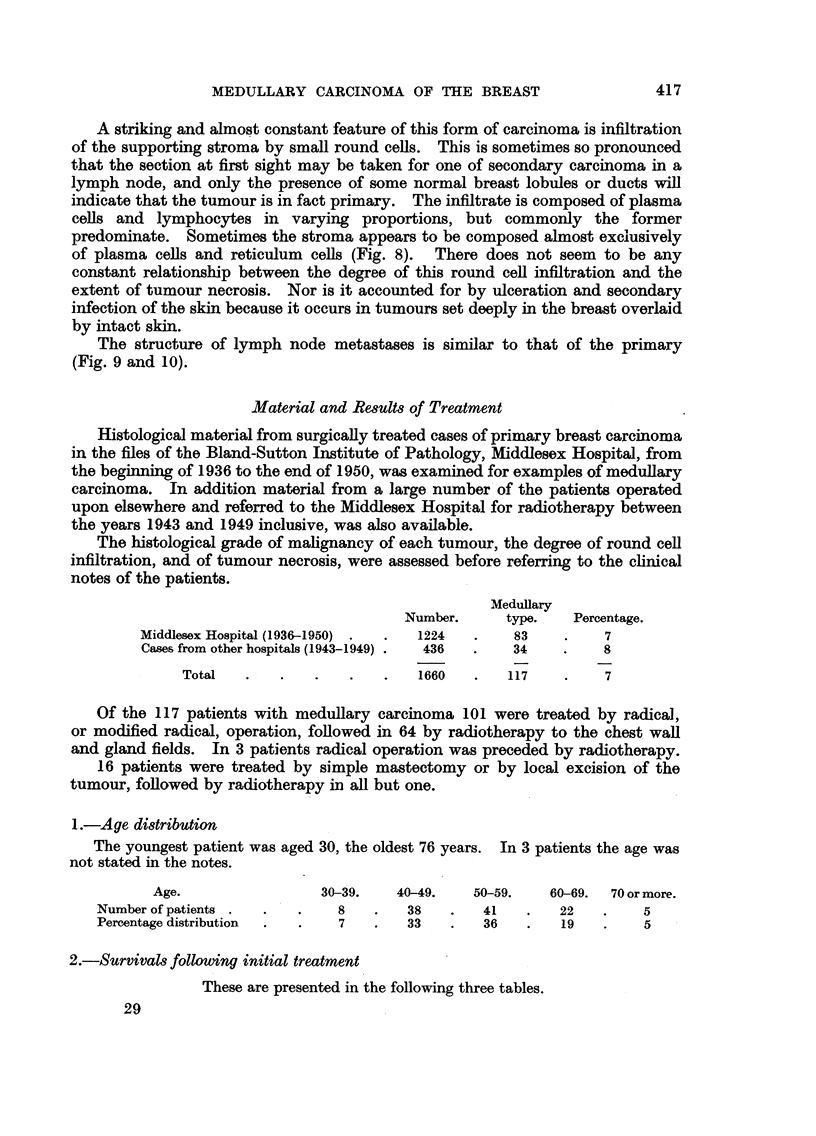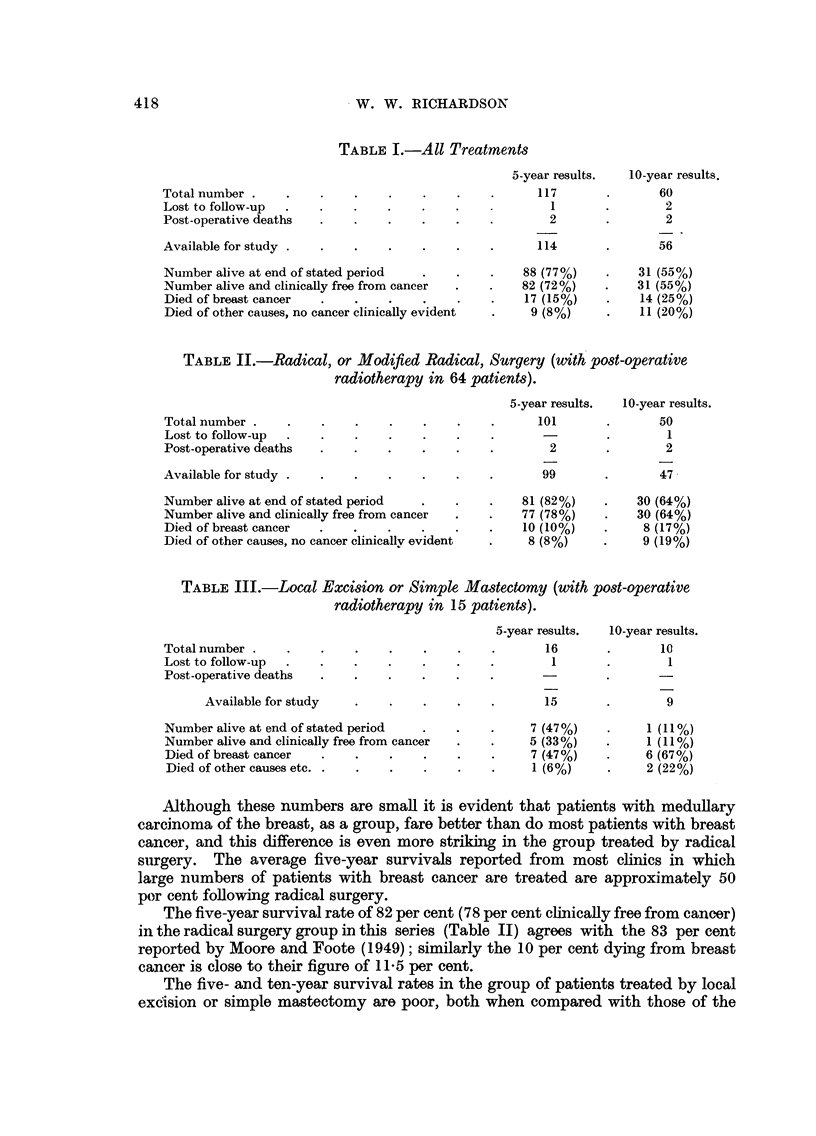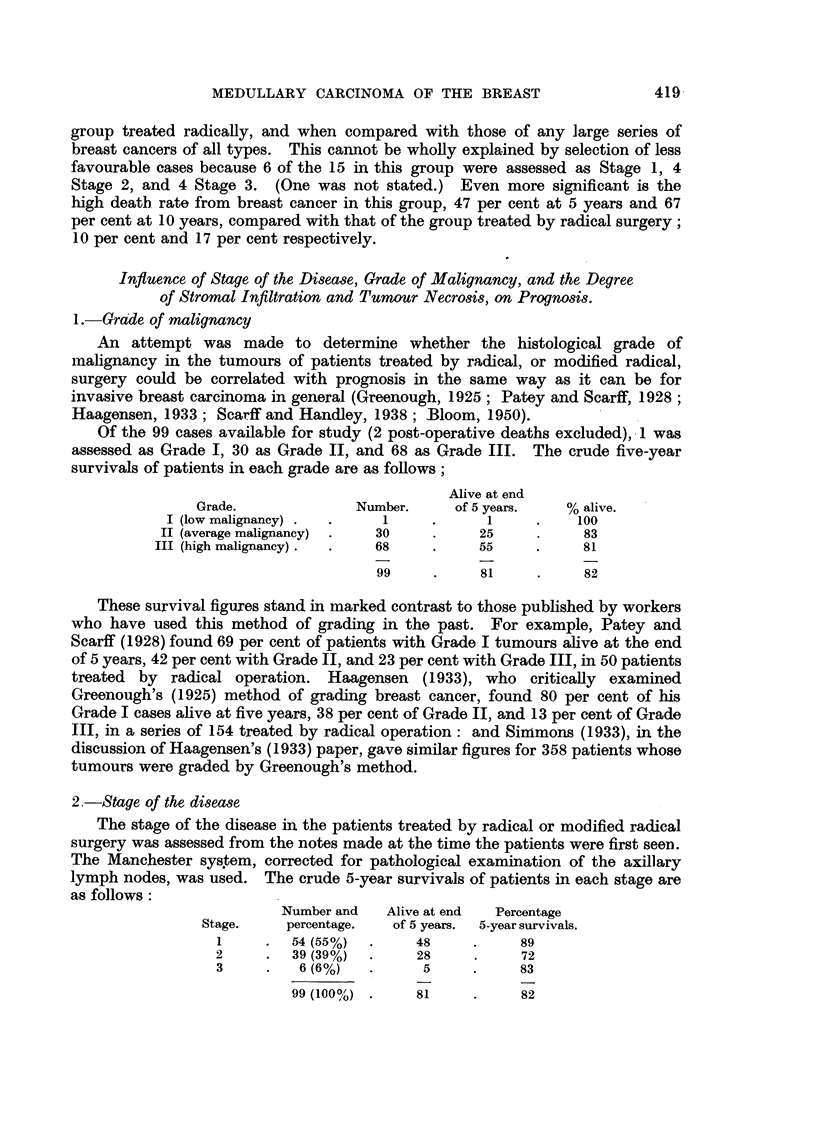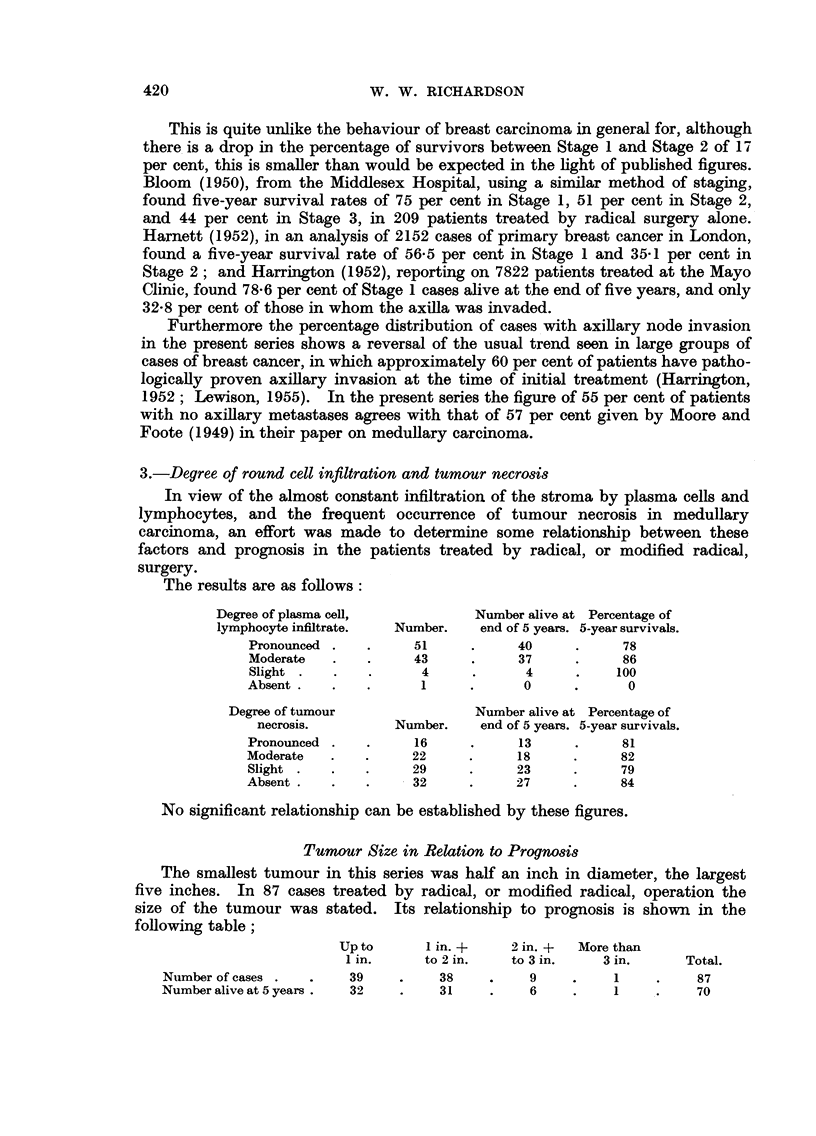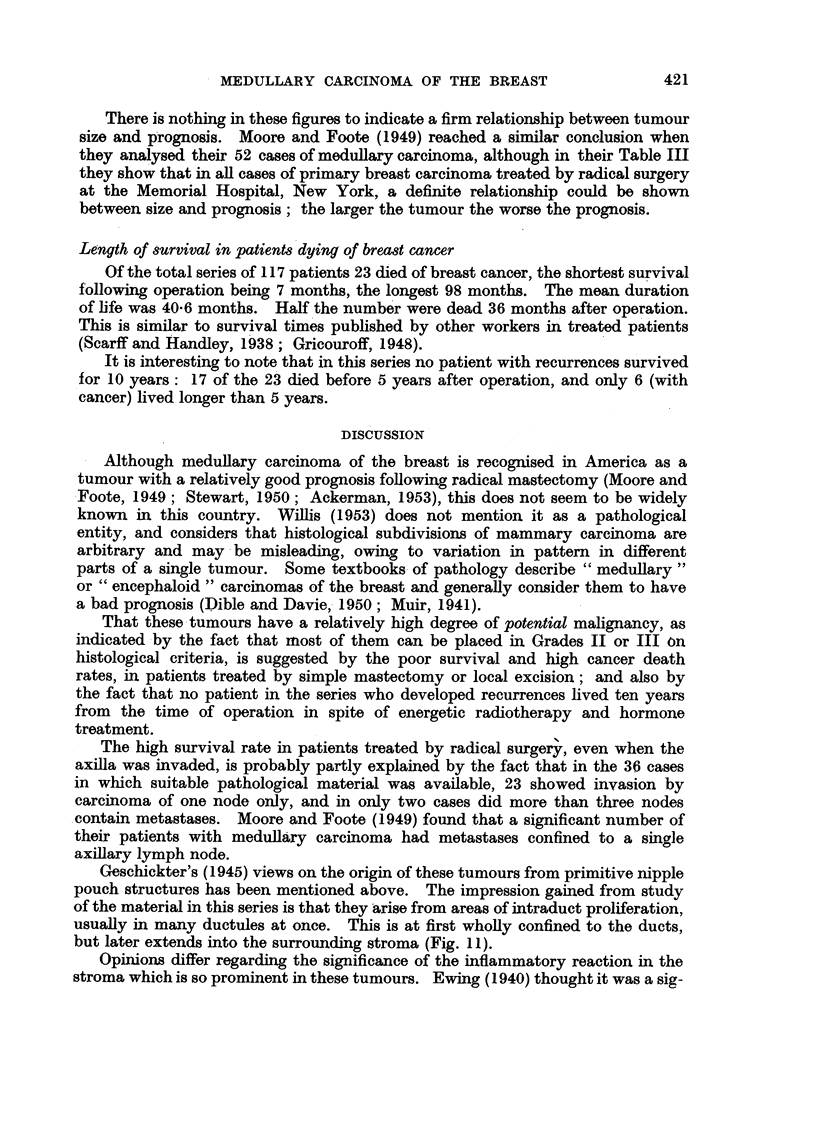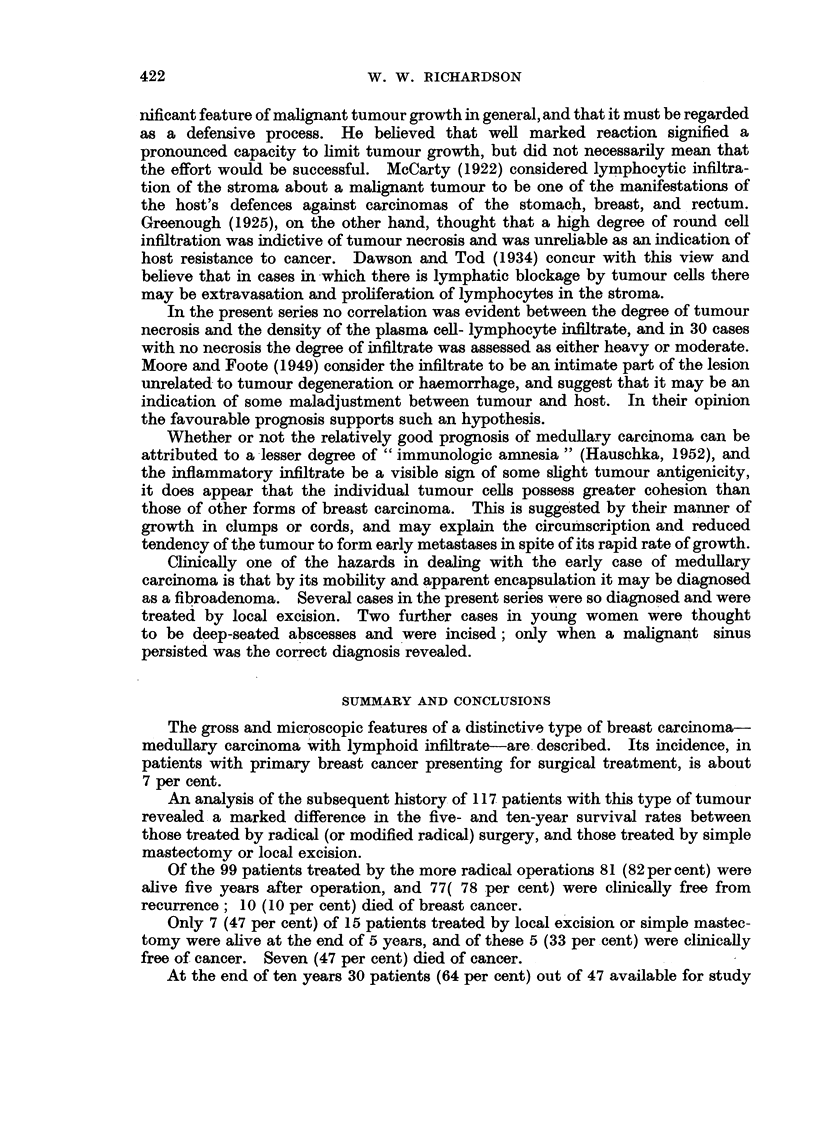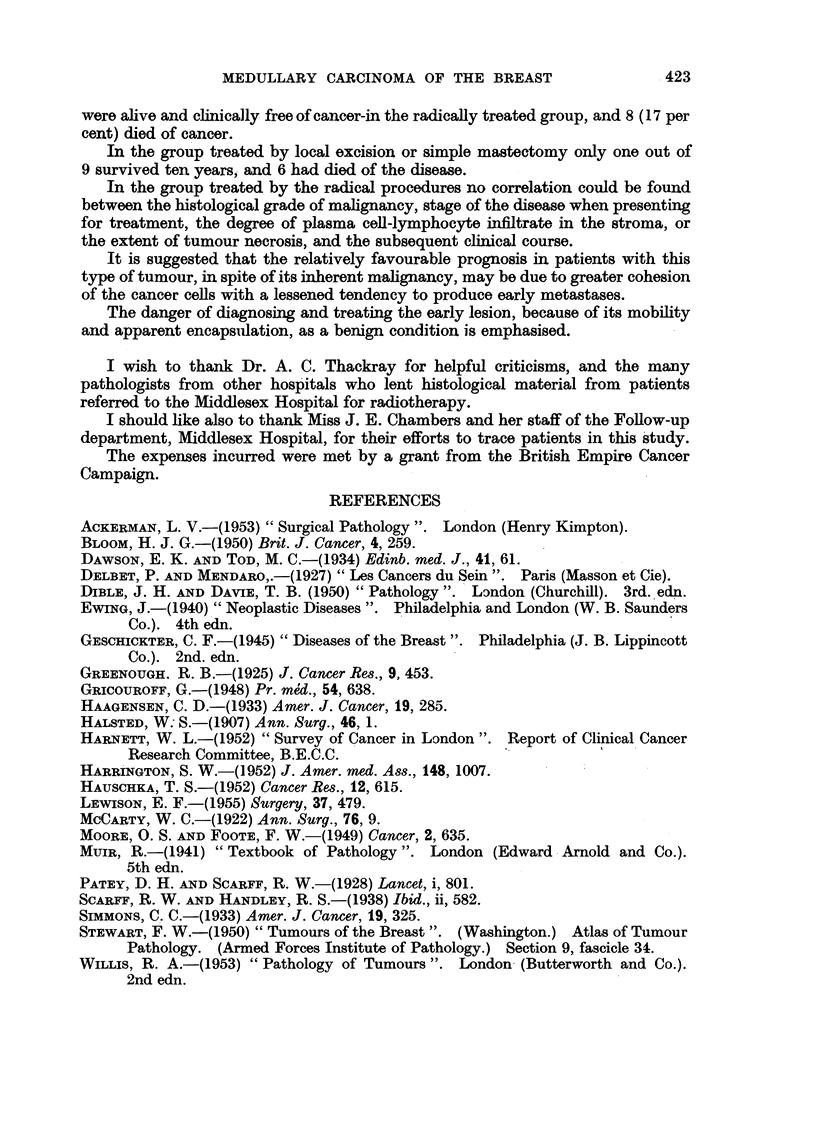# Medullary Carcinoma of the Breast

**DOI:** 10.1038/bjc.1956.48

**Published:** 1956-09

**Authors:** W. W. Richardson

## Abstract

**Images:**


### MEDULLARY CARCINOMA OF THE BREAST

### A Distinctive Tumour Type with a Relatively Good Prognosis following Radical Mastectomy

W. W. RICHARDSON

*From the Bland-Sutotn Institute of Pathology, Middlesex Hospital, London, W.1*

Many attempts have been made in the past to correlate prognosis with the histological appearances of carcinoma of the breast, with varying measures of success. The most promising results have been obtained by using the concept of *anaplasia*, first evolved by von Hansemann, which can be applied to the majority of breast cancers (Greenough, 1925 ; Patey and Scarff, 1928 ; Haagensen, 1933 ; Bloom, 1950).

With the exception of certain rare kinds, attempts to relate prognosis to definite structural tumour types, such as scirrhous, medullary, lobular, and so on, have proved disappointing and have been abandoned by most pathologists. However, most workers agree that the well-defined adenocarcinomas, with a regular acinar pattern, have a better prognosis as a group than less differentiated tumours (Halsted, 1907 ; Greenough, 1925 ; Gricouroff, 1948 ; Harrington, 1952). Similarly non-infiltrating duct carcinomas and Paget's disease of the nipple have a better prognosis in general than invasive tumours. There is less agreement on the significance of the so-called " colloid ", or " mucoid ", carcinomas, some placing them in the lowest grade of malignancy (Delbet and Mendaro, 1927 ; Haagensen, 1933 ; Gricouroff, 1948), while others do not consider that the presence of mucin has any prognostic significance (Stewart, 1950).

While studying the relationship between histological grading and prognosis in patients with breast cancer seen at the Middlesex Hospital it was noted that a certain characteristic tumour type, medullary or papillary in structure, although rated as being of high or average grade of malignancy had a better prognosis than others of the same histological grade. Geschickter (1945) described medullary cancer and cancer cysts as " neo-mammary " cancer, because he thought that they arose from the primitive nipple pouch, and he stated that the prognosis in this type of cancer is better than one would expect from the histological appearances. Moore and Foote (1949), from the Memorial Hospital, New York, clearly defined this type of tumour as " medullary carcinoma with lymphoid infiltrate ". They found 52 cases in 1000 consecutive radical mastectomy specimens, 43 (82·7 per cent) of which were alive and clinically free from cancer five years after operation.

### *Gross and Microscopic Characteristics*

The gross appearances of medullary carcinoma are usually quite distinctive. It is a spherical, well-circumsrribed tumour which may sometimes appear encapsulated. It grows by expansion rather than by infiltration which is so prominent a feature of the scirrhous type of growth (Fig. 1).

On section the tumour bulges outwards from the cut surface of the breast in contradistinction to the scirrhous form which shrinks from it. The colour is greyish-white and, in the larger specimens, yellow zones of necrosis and red or brown areas of haemorrhage are common in the centre. Occasionally the centre is cystic. The tumour tissue looks moister and more succulent than that of other types of breast carcinoma.

Microscopically the tumour has well-defined boundaries with a circumscribed growing edge, confirming the impression of expansile growth given by the gross appearance. Sometimes there appears to be a zone of compressed fibrous tissue surrounding it.

In the commonest form the tumour cells are arranged in anastomosing cords separated by a loose fibrillary connective tissue stroma (Fig. 2 and 3). The tumour cells farthest away from the stroma commonly undergo necrosis, with the result that the bands of tumour appear to surround granular eosinophilic central masses, between which and the viable cells are cancer cells in varying stages of degeneration.

Some tumours are almost wholly papillary in structure (Fig. 4), others may be composed mainly of small clumps of cells (Fig. 5) : and both these features may be seen in some parts, and solid anastomosing cords in others, of the same tumour. Shrinkage spaces occur between the cancer cells and the stroma in the ordinary paraffin-embedded sections, but in spite of this there appears to be a basement membrane separating the two. The stroma in direct contact with the basal layer of tumour cells is formed by a narrow zone of flattened spindle cells and fine collagenous fibres.

In general the tumour cells are arranged in sheets but, especially in the papillary forms, glandular acini occur. This feratue may be so pronounced as to justify the description papillary adenocarcinoma. The cells themselves may vary greatly in appearance. Most commonly they have regular spherical nuclei, 20–30 m$\mu$. in diameter, with one or two prominent nucleoli and scanty reticular chromatin (Fig. 6), but in some tumours the cells are more pleomorphic and the nuclei more variable in their staining (Fig. 7). Cytoplasm is usually abundant, stains well with eosin, and has a finely granular structure. Squamous metaplasia is rare, but occasionally giant forms are seen.

Mitotic figures are prominent ; seldom are there less than four per high power field, and abnormal forms are common.

---

EXPLANATION OF PLATES

FIG. 1.—Gross specimen of breast and axillary tissue showing a well-circumscribed medullary carcinoma with haemorrhagic area caused by biopsy incision.

FIG. 2 and 3.—Medullary carcinoma of the breast showing the characteristic structure of anastomosing cords of tumour with central necrotic zones. $\times$ 50.

FIG. 4.—Medullary carcinoma with areas of papillary growth. $\times$ 50.

FIG. 5.—Medullary carcinoma showing growth in clumps and cords of cells, and absence of tumour necrosis. $\times$ 50.

FIG. 6.—High power view of the growing edge of a medullary carcinoma. $\times$ 345.

FIG. 7.—High power of a papillary area in a medullary carcinoma in which the cells are more pleomorphic than in Fig. 6. $\times$ 345.

FIG. 8.—Growing edge of a medullary carcinoma to show the pronounced plasma-cell infiltrate in the stroma. $\times$ 400.

FIG. 9 and 10.—Low power view of a primary, which appears to be arising in a cyst, and the metastasis in an axillary node, to show the similarity of pattern. $\times$ 25.

FIG. 11.—Early medullary carcinoma showing intraduct proliferation (top) and invasive areas below. $\times$ 50.

1

2

3

4

5

Richardson.

6

7

8

9

10

11

Richardson.

A striking and almost constant feature of this form of carcinoma is infiltration of the supporting stroma by small round cells. This is sometimes so pronounced that the section at first sight may be taken for one of secondary carcinoma in a lymph node, and only the presence of some normal breast lobules or ducts will indicate that the tumour is in fact primary. The infiltrate is composed of plasma cells and lymphocytes in varying proportions, but commonly the former predominate. Sometimes the stroma appears to be composed almost exclusively of plasma cells and reticulum cells (Fig. 8). There does not seem to be any constant relationship between the degree of this round cell infiltration and the extent of tumour necrosis. Nor is it accounted for by ulceration and secondary infection of the skin because it occurs in tumours set deeply in the breast overlaid by intact skin.

The structure of lymph node metastases is similar to that of the primary (Fig. 9 and 10).

### *Material and Results of Treatment*

Histological material from surgically treated cases of primary breast carcinoma in the files of the Bland-Sutton Institute of Pathology, Middlesex Hospital, from the beginning of 1936 to the end of 1950, was examined for examples of medullary carcinoma. In addition material from a large number of the patients operated upon elsewhere and referred to the Middlesex Hospital for radiotherapy between the years 1943 and 1949 inclusive, was also available.

The histological grade of malignancy of each tumour, the degree of round cell infiltration, and of tumour necrosis, were assessed before referring to the clinical notes of the patients.

| | Number. | Medullary type. | Percentage. |
|---|---|---|---|
| Middlesex Hospital (1936–1950) | 1224 | 83 | 7 |
| Cases from other hospitals (1943–1949) | 436 | 34 | 8 |
| Total | 1660 | 117 | 7 |

Of the 117 patients with medullary carcinoma 101 were treated by radical, or modified radical, operation, followed in 64 by radiotherapy to the chest wall and gland fields. In 3 patients radical operation was preceded by radiotherapy.

16 patients were treated by simple mastectomy or by local excision of the tumour, followed by radiotherapy in all but one.

1.—*Age distribution*

The youngest patient was aged 30, the oldest 76 years. In 3 patients the age was not stated in the notes.

| Age. | 30–39. | 40–49. | 50–59. | 60–69. | 70 or more. |
|---|---|---|---|---|---|
| Number of patients | 8 | 38 | 41 | 22 | 5 |
| Percentage distribution | 7 | 33 | 36 | 19 | 5 |

2.—*Survivals following initial treatment*

These are presented in the following three tables.

TABLE I.—*All Treatments*

| | 5-year results. | 10-year results. |
|---|---|---|
| Total number | 117 | 60 |
| Lost to follow-up | 1 | 2 |
| Post-operative deaths | 2 | 2 |
| Available for study | 114 | 56 |
| Number alive at end of stated period | 88 (77%) | 31 (55%) |
| Number alive and clinically free from cancer | 82 (72%) | 31 (55%) |
| Died of breast cancer | 17 (15%) | 14 (25%) |
| Died of other causes, no cancer clinically evident | 9 (8%) | 11 (20%) |

TABLE II.—*Radical, or Modified Radical, Surgery (with post-operative radiotherapy in* 64 *patients).*

| | 5-year results. | 10-year results. |
|---|---|---|
| Total number | 101 | 50 |
| Lost to follow-up | — | 1 |
| Post-operative deaths | 2 | 2 |
| Available for study | 99 | 47 |
| Number alive at end of stated period | 81 (82%) | 30 (64%) |
| Number alive and clinically free from cancer | 77 (78%) | 30 (64%) |
| Died of breast cancer | 10 (10%) | 8 (17%) |
| Died of other causes, no cancer clinically evident | 8 (8%) | 9 (19%) |

TABLE III.—*Local Excision or Simple Mastectomy (with post-operative radiotherapy in* 15 *patients).*

| | 5-year results. | 10-year results. |
|---|---|---|
| Total number | 16 | 10 |
| Lost to follow-up | 1 | 1 |
| Post-operative deaths | — | — |
| Available for study | 15 | 9 |
| Number alive at end of stated period | 7 (47%) | 1 (11%) |
| Number alive and clinically free from cancer | 5 (33%) | 1 (11%) |
| Died of breast cancer | 7 (47%) | 6 (67%) |
| Died of other causes etc. | 1 (6%) | 2 (22%) |

Although these numbers are small it is evident that patients with medullary carcinoma of the breast, as a group, fare better than do most patients with breast cancer, and this difference is even more striking in the group treated by radical surgery. The average five-year survivals reported from most clinics in which large numbers of patients with breast cancer are treated are approximately 50 per cent following radical surgery.

The five-year survival rate of 82 per cent (78 per cent clinically free from cancer) in the radical surgery group in this series (Table II) agrees with the 83 per cent reported by Moore and Foote (1949); similarly the 10 per cent dying from breast cancer is close to their figure of 11·5 per cent.

The five- and ten-year survival rates in the group of patients treated by local excision or simple mastectomy are poor, both when compared with those of the group treated radically, and when compared with those of any large series of breast cancers of all types. This cannot be wholly explained by selection of less favourable cases because 6 of the 15 in this group were assessed as Stage 1, 4 Stage 2, and 4 Stage 3. (One was not stated.) Even more significant is the high death rate from breast cancer in this group, 47 per cent at 5 years and 67 per cent at 10 years, compared with that of the group treated by radical surgery ; 10 per cent and 17 per cent respectively.

*Influence of Stage of the Disease, Grade of Malignancy, and the Degree of Stromal Infiltration and Tumour Necrosis, on Prognosis.*

1.—*Grade of malignancy*

An attempt was made to determine whether the histological grade of malignancy in the tumours of patients treated by radical, or modified radical, surgery could be correlated with prognosis in the same way as it can be for invasive breast carcinoma in general (Greenough, 1925 ; Patey and Scarff, 1928 ; Haagensen, 1933 ; Scarff and Handley, 1938 ; Bloom, 1950).

Of the 99 cases available for study (2 post-operative deaths excluded), 1 was assessed as Grade I, 30 as Grade II, and 68 as Grade III. The crude five-year survivals of patients in each grade are as follows ;

| Grade. | Number. | Alive at end of 5 years. | % alive. |
|---|---|---|---|
| I (low malignancy) | 1 | 1 | 100 |
| II (average malignancy) | 30 | 25 | 83 |
| III (high malignancy) | 68 | 55 | 81 |
| | 99 | 81 | 82 |

These survival figures stand in marked contrast to those published by workers who have used this method of grading in the past. For example, Patey and Scarff (1928) found 69 per cent of patients with Grade I tumours alive at the end of 5 years, 42 per cent with Grade II, and 23 per cent with Grade III, in 50 patients treated by radical operation. Haagensen (1933), who critically examined Greenough's (1925) method of grading breast cancer, found 80 per cent of his Grade I cases alive at five years, 38 per cent of Grade II, and 13 per cent of Grade III, in a series of 154 treated by radical operation : and Simmons (1933), in the discussion of Haagensen's (1933) paper, gave similar figures for 358 patients whose tumours were graded by Greenough's method.

2.—*Stage of the disease*

The stage of the disease in the patients treated by radical or modified radical surgery was assessed from the notes made at the time the patients were first seen. The Manchester system, corrected for pathological examination of the axillary lymph nodes, was used. The crude 5-year survivals of patients in each stage are as follows :

| Stage. | Number and percentage. | Alive at end of 5 years. | Percentage 5-year survivals. |
|---|---|---|---|
| 1 | 54 (55%) | 48 | 89 |
| 2 | 39 (39%) | 28 | 72 |
| 3 | 6 (6%) | 5 | 83 |
| | 99 (100%) | 81 | 82 |

This is quite unlike the behaviour of breast carcinoma in general for, although there is a drop in the percentage of survivors between Stage 1 and Stage 2 of 17 per cent, this is smaller than would be expected in the light of published figures. Bloom (1950), from the Middlesex Hospital, using a similar method of staging, found five-year survival rates of 75 per cent in Stage 1, 51 per cent in Stage 2, and 44 per cent in Stage 3, in 209 patients treated by radical surgery alone. Harnett (1952), in an analysis of 2152 cases of primary breast cancer in London, found a five-year survival rate of 56·5 per cent in Stage 1 and 35·1 per cent in Stage 2 ; and Harrington (1952), reporting on 7822 patients treated at the Mayo Clinic, found 78·6 per cent of Stage 1 cases alive at the end of five years, and only 32·8 per cent of those in whom the axilla was invaded.

Furthermore the percentage distribution of cases with axillary node invasion in the present series shows a reversal of the usual trend seen in large groups of cases of breast cancer, in which approximately 60 per cent of patients have pathologically proven axillary invasion at the time of initial treatment (Harrington, 1952 ; Lewison, 1955). In the present series the figure of 55 per cent of patients with no axillary metastases agrees with that of 57 per cent given by Moore and Foote (1949) in their paper on medullary carcinoma.

3.—*Degree of round cell infiltration and tumour necrosis*

In view of the almost constant infiltration of the stroma by plasma cells and lymphocytes, and the frequent occurrence of tumour necrosis in medullary carcinoma, an effort was made to determine some relationship between these factors and prognosis in the patients treated by radical, or modified radical, surgery.

The results are as follows :

| Degree of plasma cell, lymphocyte infiltrate. | Number. | Number alive at end of 5 years. | Percentage of 5-year survivals. |
|---|---|---|---|
| Pronounced | 51 | 40 | 78 |
| Moderate | 43 | 37 | 86 |
| Slight | 4 | 4 | 100 |
| Absent | 1 | 0 | 0 |

| Degree of tumour necrosis. | Number. | Number alive at end of 5 years. | Percentage of 5-year survivals. |
|---|---|---|---|
| Pronounced | 16 | 13 | 81 |
| Moderate | 22 | 18 | 82 |
| Slight | 29 | 23 | 79 |
| Absent | 32 | 27 | 84 |

No significant relationship can be established by these figures.

### *Tumour Size in Relation to Prognosis*

The smallest tumour in this series was half an inch in diameter, the largest five inches. In 87 cases treated by radical, or modified radical, operation the size of the tumour was stated. Its relationship to prognosis is shown in the following table ;

| | Up to 1 in. | 1 in. + to 2 in. | 2 in. + to 3 in. | More than 3 in. | Total. |
|---|---|---|---|---|---|
| Number of cases | 39 | 38 | 9 | 1 | 87 |
| Number alive at 5 years | 32 | 31 | 6 | 1 | 70 |

There is nothing in these figures to indicate a firm relationship between tumour size and prognosis. Moore and Foote (1949) reached a similar conclusion when they analysed their 52 cases of medullary carcinoma, although in their Table III they show that in all cases of primary breast carcinoma treated by radical surgery at the Memorial Hospital, New York, a definite relationship could be shown between size and prognosis ; the larger the tumour the worse the prognosis.

*Length of survival in patients dying of breast cancer*

Of the total series of 117 patients 23 died of breast cancer, the shortest survival following operation being 7 months, the longest 98 months. The mean duration of life was 40·6 months. Half the number were dead 36 months after operation. This is similar to survival times published by other workers in treated patients (Scarff and Handley, 1938 ; Gricouroff, 1948).

It is interesting to note that in this series no patient with recurrences survived for 10 years : 17 of the 23 died before 5 years after operation, and only 6 (with cancer) lived longer than 5 years.

### DISCUSSION

Although medullary carcinoma of the breast is recognised in America as a tumour with a relatively good prognosis following radical mastectomy (Moore and Foote, 1949 ; Stewart, 1950 ; Ackerman, 1953), this does not seem to be widely known in this country. Willis (1953) does not mention it as a pathological entity, and considers that histological subdivisions of mammary carcinoma are arbitrary and may be misleading, owing to variation in pattern in different parts of a single tumour. Some textbooks of pathology describe " medullary " or " encephaloid " carcinomas of the breast and generally consider them to have a bad prognosis (Dible and Davie, 1950 ; Muir, 1941).

That these tumours have a relatively high degree of *potential* malignancy, as indicated by the fact that most of them can be placed in Grades II or III on histological criteria, is suggested by the poor survival and high cancer death rates, in patients treated by simple mastectomy or local excision ; and also by the fact that no patient in the series who developed recurrences lived ten years from the time of operation in spite of energetic radiotherapy and hormone treatment.

The high survival rate in patients treated by radical surgery, even when the axilla was invaded, is probably partly explained by the fact that in the 36 cases in which suitable pathological material was available, 23 showed invasion by carcinoma of one node only, and in only two cases did more than three nodes contain metastases. Moore and Foote (1949) found that a significant number of their patients with medullary carcinoma had metastases confined to a single axillary lymph node.

Geschickter's (1945) views on the origin of these tumours from primitive nipple pouch structures has been mentioned above. The impression gained from study of the material in this series is that they arise from areas of intraduct proliferation, usually in many ductules at once. This is at first wholly confined to the ducts, but later extends into the surrounding stroma (Fig. 11).

Opinions differ regarding the significance of the inflammatory reaction in the stroma which is so prominent in these tumours. Ewing (1940) thought it was a significant feature of malignant tumour growth in general, and that it must be regarded as a defensive process. He believed that well marked reaction signified a pronounced capacity to limit tumour growth, but did not necessarily mean that the effort would be successful. McCarty (1922) considered lymphocytic infiltration of the stroma about a malignant tumour to be one of the manifestations of the host's defences against carcinomas of the stomach, breast, and rectum. Greenough (1925), on the other hand, thought that a high degree of round cell infiltration was indictive of tumour necrosis and was unreliable as an indication of host resistance to cancer. Dawson and Tod (1934) concur with this view and believe that in cases in which there is lymphatic blockage by tumour cells there may be extravasation and proliferation of lymphocytes in the stroma.

In the present series no correlation was evident between the degree of tumour necrosis and the density of the plasma cell- lymphocyte infiltrate, and in 30 cases with no necrosis the degree of infiltrate was assessed as either heavy or moderate. Moore and Foote (1949) consider the infiltrate to be an intimate part of the lesion unrelated to tumour degeneration or haemorrhage, and suggest that it may be an indication of some maladjustment between tumour and host. In their opinion the favourable prognosis supports such an hypothesis.

Whether or not the relatively good prognosis of medullary carcinoma can be attributed to a lesser degree of " immunologic amnesia " (Hauschka, 1952), and the inflammatory infiltrate be a visible sign of some slight tumour antigenicity, it does appear that the individual tumour cells possess greater cohesion than those of other forms of breast carcinoma. This is suggested by their manner of growth in clumps or cords, and may explain the circumscription and reduced tendency of the tumour to form early metastases in spite of its rapid rate of growth.

Clinically one of the hazards in dealing with the early case of medullary carcinoma is that by its mobility and apparent encapsulation it may be diagnosed as a fibroadenoma. Several cases in the present series were so diagnosed and were treated by local excision. Two further cases in young women were thought to be deep-seated abscesses and were incised ; only when a malignant sinus persisted was the correct diagnosis revealed.

### SUMMARY AND CONCLUSIONS

The gross and microscopic features of a distinctive type of breast carcinoma—medullary carcinoma with lymphoid infiltrate—are described. Its incidence, in patients with primary breast cancer presenting for surgical treatment, is about 7 per cent.

An analysis of the subsequent history of 117 patients with this type of tumour revealed a marked difference in the five- and ten-year survival rates between those treated by radical (or modified radical) surgery, and those treated by simple mastectomy or local excision.

Of the 99 patients treated by the more radical operations 81 (82 per cent) were alive five years after operation, and 77( 78 per cent) were clinically free from recurrence ; 10 (10 per cent) died of breast cancer.

Only 7 (47 per cent) of 15 patients treated by local excision or simple mastectomy were alive at the end of 5 years, and of these 5 (33 per cent) were clinically free of cancer. Seven (47 per cent) died of cancer.

At the end of ten years 30 patients (64 per cent) out of 47 available for study were alive and clinically free of cancer in the radically treated group, and 8 (17 per cent) died of cancer.

In the group treated by local excision or simple mastectomy only one out of 9 survived ten years, and 6 had died of the disease.

In the group treated by the radical procedures no correlation could be found between the histological grade of malignancy, stage of the disease when presenting for treatment, the degree of plasma cell-lymphocyte infiltrate in the stroma, or the extent of tumour necrosis, and the subsequent clinical course.

It is suggested that the relatively favourable prognosis in patients with this type of tumour, in spite of its inherent malignancy, may be due to greater cohesion of the cancer cells with a lessened tendency to produce early metastases.

The danger of diagnosing and treating the early lesion, because of its mobility and apparent encapsulation, as a benign condition is emphasised.

I wish to thank Dr. A. C. Thackray for helpful criticisms, and the many pathologists from other hospitals who lent histological material from patients referred to the Middlesex Hospital for radiotherapy.

I should like also to thank Miss J. E. Chambers and her staff of the Follow-up department, Middlesex Hospital, for their efforts to trace patients in this study.

The expenses incurred were met by a grant from the British Empire Cancer Campaign.

### REFERENCES

Ackerman, L. V.—(1953) " Surgical Pathology ". London (Henry Kimpton).
Bloom, H. J. G.—(1950) *Brit. J. Cancer*, **4**, 259.
Dawson, E. K. and Tod, M. C.—(1934) *Edinb. med. J.*, **41**, 61.
Delbet, P. and Mendaro,.—(1927) " Les Cancers du Sein ". Paris (Masson et Cie).
Dible, J. H. and Davie, T. B. (1950) " Pathology ". London (Churchill). 3rd. edn.
Ewing, J.—(1940) " Neoplastic Diseases ". Philadelphia and London (W. B. Saunders Co.). 4th edn.
Geschickter, C. F.—(1945) " Diseases of the Breast ". Philadelphia (J. B. Lippincott Co.). 2nd. edn.
Greenough. R. B.—(1925) *J. Cancer Res.*, **9**, 453.
Gricouroff, G.—(1948) *Pr. méd.*, **54**, 638.
Haagensen, C. D.—(1933) *Amer. J. Cancer*, **19**, 285.
Halsted, W. S.—(1907) *Ann. Surg.*, **46**, 1.
Harnett, W. L.—(1952) " Survey of Cancer in London ". Report of Clinical Cancer Research Committee, B.E.C.C.
Harrington, S. W.—(1952) *J. Amer. med. Ass.*, **148**, 1007.
Hauschka, T. S.—(1952) *Cancer Res.*, **12**, 615.
Lewison, E. F.—(1955) *Surgery*, **37**, 479.
McCarty, W. C.—(1922) *Ann. Surg.*, **76**, 9.
Moore, O. S. and Foote, F. W.—(1949) *Cancer*, **2**, 635.
Muir, R.—(1941) " Textbook of Pathology ". London (Edward Arnold and Co.). 5th edn.
Patey, D. H. and Scarff, R. W.—(1928) *Lancet*, i, 801.
Scarff, R. W. and Handley, R. S.—(1938) *Ibid.*, ii, 582.
Simmons, C. C.—(1933) *Amer. J. Cancer*, **19**, 325.
Stewart, F. W.—(1950) " Tumours of the Breast ". (Washington.) Atlas of Tumour Pathology. (Armed Forces Institute of Pathology.) Section 9, fascicle 34.
Willis, R. A.—(1953) " Pathology of Tumours ". London (Butterworth and Co.). 2nd edn.